# Supplementary material for: Identification and Functional Analysis of NLP-Encoding Genes from the Postharvest Pathogen Penicillium expansum
Source: Microorganisms. 2019 Jun 15;7(6):175. doi: 10.3390/microorganisms7060175 (PMC6616513; doi:10.3390/microorganisms7060175)
Supplement: Supplementary file 1 [file microorganisms-07-00175-s001.pdf]

Supplementary Information

Identification and Functional Analysis of NLP-Encoding Genes from the Postharvest Pathogen *Penicillium expansum*

Table S1. Primers used in the study.

	Name	Sequence	Explanation
1	NLP1_ center_F	TTT CCA GCT CCA AAG TCG	testing of deletion of *PeNLP1*
2	NLP1_ center_R	TAT TGA CTA ACG GGC TTC CT	testing of deletion of *PeNLP1*
3	HMBR1	CTG ATA GAG TTG GTC AAG ACC	insertion of the selection marker
4	HMBF1	CTG TCG AGA AGT TTC TGA TCG	insertion of the selection marker
5	NLP1_checkUP	GGT TGA GAT TCC CCA CAC GAT AGC	upstream integration of *PeNLP1*
6	HPH1F	ACG AGG TCG CCA ACA TCT TCT TCT	upstream integration of the T-DNA
7	HPHPRO4	GCA CCA AGC AGC AGA TGA TA	downstream integration of the T-DNA
8	NLP1_checkDOWN	TGC CCG TTC CGA ATG A	downstream integration of *PeNLP1*
9	NLP2_ center_F	ACC GGG CAT GAC TAG TAG GT	testing of deletion of *PeNLP2*
10	NLP2_ center_R	TGC ACT ACC CCA ATC AGC	testing of deletion of *PeNLP2*
11	NLP2_checkUP	GCT TGA CTA TCT CCT AAT TGG TGG GC	upstream integration of the *PeNLP2*
12	NLP2_checkDOWN	AGT TCT AGA CGG ACT TGC GG	downstream integration of *PeNLP2*
13	NLP1_CN_F	CGG ACC ATC TTT ATG CGT GA	determination of T-DNA copy number
14	NLP1_CN_R	GAA CAA ATG CTA CTG CCA ATG C	determination of T-DNA copy number
15	NLP2_CNP_F2	GCC GGT TCC ATT TCA AAG CA	determination of T-DNA copy number
16	NLP2_CNP_R2	TGA TCA TTC CAG AGC CCG TT	determination of T-DNA copy number
17	PeTub_F	AGC GGT GAC AAG TAC GTT CC	determination of T-DNA copy number
18	PeTub_R	ACC CTT AGC CCA GTT GTT AC	determination of T-DNA copy number
19	NLP1_Prom_F	GGT CTT AAU CAG CAG GTG TCT TGG GTA C	5′ flanking region *PeNLP1*
20	NLP1_Prom_R	GGC ATT AAU GAT CAA CCT TCA TAT AAT GCT CTC	5′ flanking region *PeNLP1*
21	NLP1_Term_F	GGA CTT AAU TAG TGG AGG CTA GTA AAG TTC G	3′ flanking region *PeNLP1*

22	NLP1_Term_R	GGG TTT AAU ATC TCG GTA CTT TTG CTA GTC C	3′ flanking region *PeNLP1*
23	NLP2_Prom_F	GGT CTT AAU GCC AAC GAA GTC AAG ACT G	5′ flanking region *PeNLP2*
24	NLP2_Prom_R	GGC ATT AAU ATC ACA GTC GTC AAG CTG C	5′ flanking region *PeNLP2*
25	NLP2_Term_F	GGA CTT AAU ATC TGG TCA CTG GCA TTC TC	3′ flanking region *PeNLP2*
26	NLP2_Term_R	GGG TTT AAU GGA CGT TGG TAT AAC TAT AGG TAG ACT	3′ flanking region *PeNLP2*
27	NLP1_complete_F	ATG CTT CCT CAA CTA ATT ACT GC	*PeNLP1* ORF
28	NLP1_complete_R	TCA GTA GGT TGC CTT TGC TAG	*PeNLP1* ORF
29	NLP2_complete_F	ATG CAC CTA AAG CTA GGA GCT AG	*PeNLP2* ORF
30	NLP2_complete_R	CTA TGA TGC CTT ACA CAC ACC A	*PeNLP2* ORF
31	NLP1_RT_F	GAT GCA AAC GGA AAC ACG AA	*PeNLP1* relative expression qRT-PCR
32	NLP1_RT_R	ACT TGC CCA GTG CTC GAA CT	*PeNLP1* relative expression qRT-PCR
33	NLP2_RT_F3	GAA TCA GCC ACC CTG CTA TCC	*PeNLP2* relative expression qRT-PCR
34	NLP2_RT_R3	TCC TGT GGG CAT CGT CAT ATA C	*PeNLP2* relative expression qRT-PCR
35	28S_F	GGA ACG GGA CGT CAT AGA GG	qRT-PCR endogenous control
36	28S_R	AGA GCT GCA TTC CCA AAC AAC	qRT-PCR endogenous control
37	37S_F	GCT CTG GTC TAC GAC TCC TC	qRT-PCR endogenous control
38	37S_R	GGA AGC CTT CTC GAT CTT GC	qRT-PCR endogenous control
39	His3_F2	TCT CCG CTT CCA GTC CTC TG	qRT-PCR endogenous control
40	His3_R2	TTG GTG TCC TCG AAG AGA GAG AC	qRT-PCR endogenous control
41	F_BamHI_SP_NLP1	AGTCGGATCCATGCTTCCTCAACTAATTACTGC	*PeNLP1* ORF for cloning into pRTL2
42	R_XbaI_NLP1	AGTCTCTAGATCAGTAGGTTGCCTTTGCTAG	*PeNLP1* ORF for cloning into pRTL2
43	F_BamHI_NS_NLP1	AGTCGGATCCATGAGCCCGCTTGAGTTGTC	*PeNLP1* ORF without signal peptide for cloning into pRTL2
44	F_BamHI_SP_NLP2	AGTCGGATCCATGCACCTAAAGCTAGGAGCTAG	*PeNLP2* ORF for cloning into pRTL2
45	R_XbaI_NLP2	AGTCTCTAGACTATGATGCCTTACACACACCA	*PeNLP2* ORF for cloning into pRTL2
46	F_BamHI_NS_NLP2	AGTCGGATCCATGGAGATGGGCGCACTCTTG	*PeNLP2* ORF without signal peptide for cloning into pRTL2
47	NLP1_Forward	CATGGAGGCCGAATTCATGCTTCCTCAACTAATTACTGCG	*PeNLP1* for bait plasmid construction
48	NLP1_Reverse	GCAGGTCGACGGATCCTCAGTAGGTTGCCTTTGCTAGGTT	*PeNLP1* for bait plasmid construction

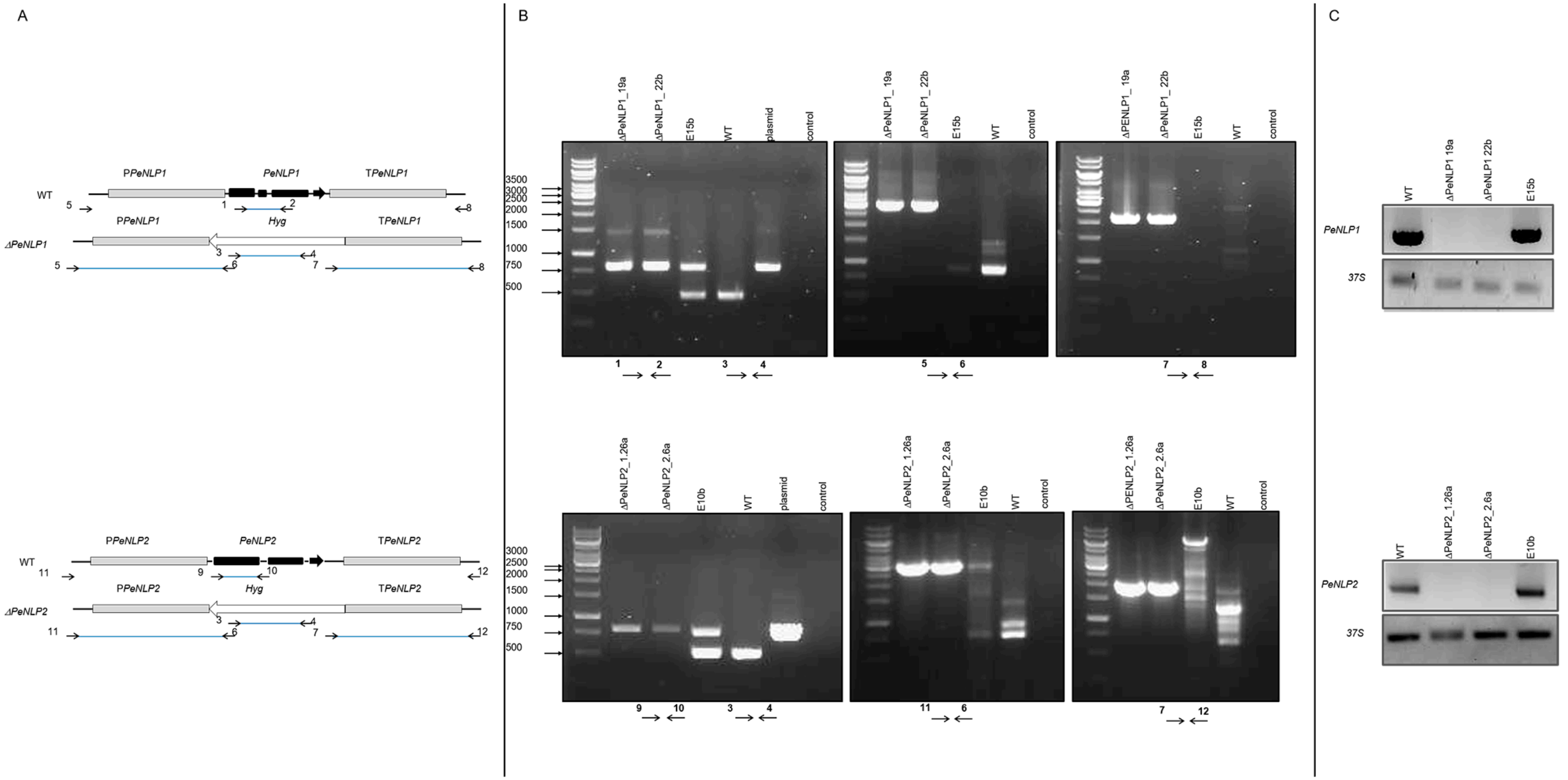

Figure S1: Deletion of *Penlp1* and *Penlp2* in *P. expansum* PEX2

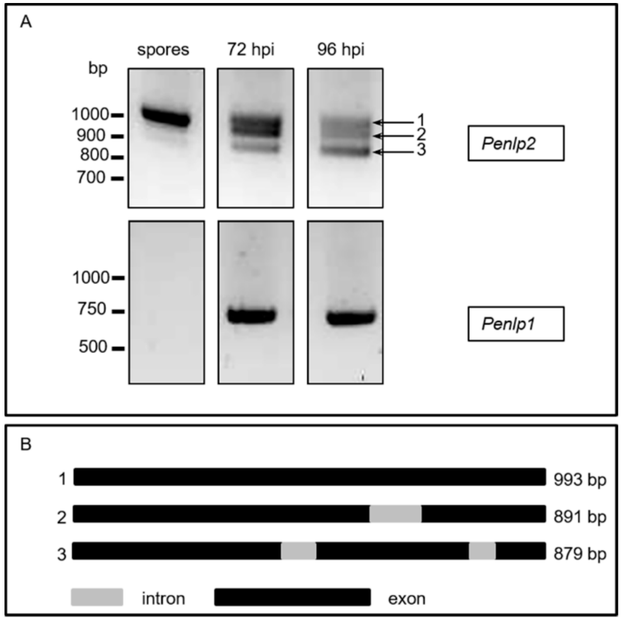

Figure S2. (**A**) Three splice variants of the *Penlp2* gene. Spore suspensions of *P. expansum* were inoculated into 100 mL of potato dextrose broth (PDB) and were incubated for 3 to 4 days at 25 °C in the dark. Full lengths ORF of *Penlp1* and *Penlp2* was amplified using PCR reaction and separated on agarose gel. Figure S2 (**B**) Schematic representation of the tree splice variants.

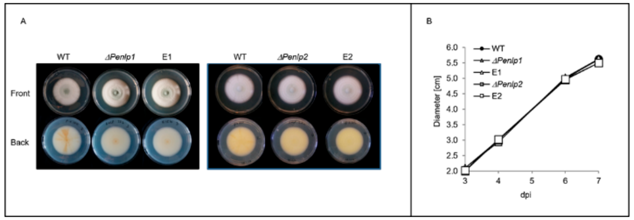

Figure S3. Targeted deletion of *Penlp1* or *Penlp2* genes does not affect radial growth and colony morphology

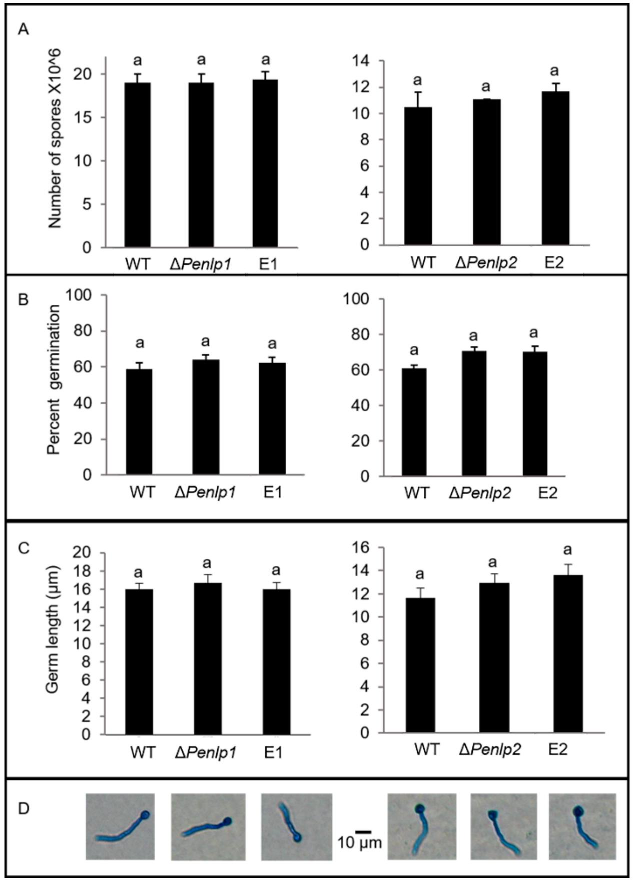

Figure S4. Targeted deletion of *Penlp1* or *Penlp2* genes had no significant effect on spore-production or germination
